# Association between psychiatric disorders and the risk of stroke: a meta-analysis of cohort studies

**DOI:** 10.3389/fneur.2024.1444862

**Published:** 2024-12-18

**Authors:** Zhonghou Hu, Weishan Sun, Enxiu Cui, Bo Chen, Mi Zhang

**Affiliations:** Third Department of Psychiatry, Yancheng Fourth People's Hospital, Yancheng, China

**Keywords:** psychiatric disorders, stroke, meta-analysis, cohort studies, risk

## Abstract

**Background:**

Psychiatric disorders may be associated with an elevated risk of stroke; however, the existence of variations in this association between different populations remains controversial. Consequently, we conducted a comprehensive systematic review and meta-analysis to examine the magnitude of the relationship between psychiatric disorders and the risk of stroke.

**Methods:**

The PubMed, Embase, and Cochrane Library databases were systematically searched to identify eligible studies from inception to April 2024. The aggregated findings were expressed as relative risks (RRs) with 95% confidence intervals (CIs), and the combined analysis was performed using a random-effects modeling approach. Further exploratory analyses were also performed using sensitivity and subgroup analyses.

**Results:**

A total of 36 cohort studies, involving 25,519,635 individuals, were selected for analysis. We noted that depression (RR: 1.50; 95% CI: 1.34–1.68; *p* < 0.001), schizophrenia (RR: 1.74; 95% CI: 1.36–2.24; *p* < 0.001), and bipolar disorder (RR: 1.65; 95% CI: 1.27–2.14; *p* < 0.001) were associated with an elevated risk of stroke. Further exploratory analyses found that the association between depression and the risk of stroke differed according to the adjusted level (RR ratio: 0.77; 95% CI: 0.61–0.98; *p* = 0.034), and the association between schizophrenia and the risk of stroke differed according to the outcome definition (RR ratio: 0.68; 95% CI: 0.52–0.90; *p* = 0.006). Moreover, the association between bipolar disorder and the risk of stroke differed according to the study design (RR ratio: 0.68; 95% CI: 0.55–0.84; *p* < 0.001).

**Conclusion:**

The significant association between psychiatric disorders and an elevated risk of stroke highlights the importance of enhanced monitoring and stroke prevention in patients with psychiatric disorders.

**Systematic review registration:**

Our study was registered on the INPLASY platform (number: INPLASY202450049).

## Introduction

By 2019, stroke remained the third leading cause of disability and the second leading cause of death globally due to its high incidence, disability rates, and mortality rates (1). Furthermore, stroke imposes a huge economic burden, leading to substantial healthcare expenditures and significant global impacts (2). Guidelines and recommendations for secondary stroke prevention at the national and international levels depend heavily on lifestyle changes (3). A previous study has shown that 90% of strokes are caused by 10 modifiable risk factors, either individually or in combination (4). By adopting lifestyle choices such as healthy eating, physical activity, maintaining normal body weight, moderate alcohol consumption, and avoiding smoking, stroke patients can lower their cardiovascular mortality rate by up to 92% over the next 10 years (5). Stroke patients typically have uncontrolled modifiable risk factors. In addition, the presence of multiple comorbidities is concerning as it is significantly associated with the prognosis of stroke patients (6).

Psychiatric disorders are a common issue in the global healthcare system. These disorders are considered a major cause of disability, contributing to 14% of the global disease burden (7). In some high-income countries, it is estimated that 40% of disabilities are caused by mental disorders (8). Psychiatric disorders are also common in the working population and are increasingly recognized due to their impact on the productivity of both employees and organizations (7). According to earlier reports, individuals diagnosed with severe psychiatric disorders (such as schizophrenia and bipolar disorder) have a life expectancy that is 15–20 years shorter than that of the general population, with cardiovascular disease being a significant cause of death among them (9–11). The relationship between psychiatric disorders and stroke is bidirectional. On the one hand, individuals with psychiatric disorders may have an increased risk of stroke, partly due to the impact of their mental health condition on other aspects of their physical health. On the other hand, stroke itself can lead to psychiatric disorders, as it affects the quality of life and psychological state of patients (12). To delve deeper into the potential causality between psychiatric disorders and the occurrence and development of stroke, we performed this systematic review and meta-analysis to assess the association between psychiatric disorders and the risk of stroke based on cohort studies.

## Methods

### Data sources, search strategy, and selection criteria

Based on the Preferred Reporting Items for Systematic Reviews and Meta-Analyses (PRISMA) guidelines published in 2020, this review adhered to and reported the recommended items (13). Our study was registered on the INPLASY platform (number: INPLASY202450049). This meta-analysis included any cohort studies that examined the relationship between psychiatric disorders and the risk of stroke, without restrictions on language or publication status (published, in press, or ongoing). Studies were included if they met the following criteria: (1) Participants comprised those who were stroke-free at the start of the study; (2) The exposure variable included psychiatric disorders, such as bipolar disorder, schizophrenia, and depression; (3) controls consisted of individuals without psychiatric disorders; (4) outcomes compared the risk of stroke between individuals with and without psychiatric disorders; and (5) the study design necessitated a cohort structure for all included studies.

We retrieved articles that were published between inception and April 2024 from the PubMed, Embase, and Cochrane Library electronic databases using the search terms “bipolar disorder” OR “schizophrenia” OR “depression” AND (“stroke risk” OR “risk of stroke”). The details of the search strategy in each database is shown in Supplementary material 1. In addition, we meticulously examined the bibliographies of all relevant primary and review articles to identify further studies that met our criteria. The selection of the relevant studies was based on medical subject headings, research methodologies, patient cohorts, study design, specified exposures, and resultant outcome measures. The literature search was carried out independently by two authors using standardized methodologies. Any discrepancies encountered were resolved by the corresponding author until mutual agreement was achieved.

### Data collection and quality assessment

The two authors extracted key information from the independent studies, including the study group name, publication year, study design, geographical region, study period, sample size, age range or mean age, male proportion, exposure and assessment methods, reported outcomes, follow-up duration, adjusted factors, and effect estimates with 95% confidence intervals (CIs). For studies reporting several multivariable-adjusted effect estimates, we selected the effect estimate with the maximal adjustment for potential confounders. The two authors then used the Newcastle–Ottawa Scale (NOS) for methodological quality assessment, which has been partially validated for the quality assessment of observational studies in meta-analyses (14). Any inconsistencies between the authors regarding the data collection and quality assessment were resolved by an additional author, who referred to the original studies.

### Statistical analysis

We analyzed the association between psychiatric disorders and the risk of stroke based on the effect estimates and their 95% CIs reported in each study. As this study’s analysis was based on a cohort design, we used the relative risk (RR) as the pooled effect estimate, and all analyses were conducted using a random-effects model to account for potential heterogeneity across the studies (15, 16). We used *I^2^* and Q statistics to analyze potential heterogeneity among the studies. Heterogeneity was considered significant if *I^2^* ≥ 50% or *p* < 0.10 (17, 18). We also systematically excluded each study from the meta-analysis and conducted a sensitivity analysis to assess the stability of the pooled conclusions and investigate the potential sources of heterogeneity (19). Subgroup analyses were also performed based on study design, geographical region, mean age, sex, stroke type, reported outcomes, follow-up duration, and adjusted levels, and the differences between the subgroups were compared using the interaction *t-*test, assuming the data followed a normal distribution (20). Publication bias was assessed using both qualitative and quantitative methods, including visual inspection of funnel plot symmetry, Egger’s test, and Begg’s test (21, 22). All reported *p*-values were two-sided, and a *p*-value of <0.05 was considered statistically significant for the meta-analysis. STATA software (version 12.0; Stata Corp, College Station, TX, USA) was used for the analysis.

## Results

### Literature search

**Figure 1:**
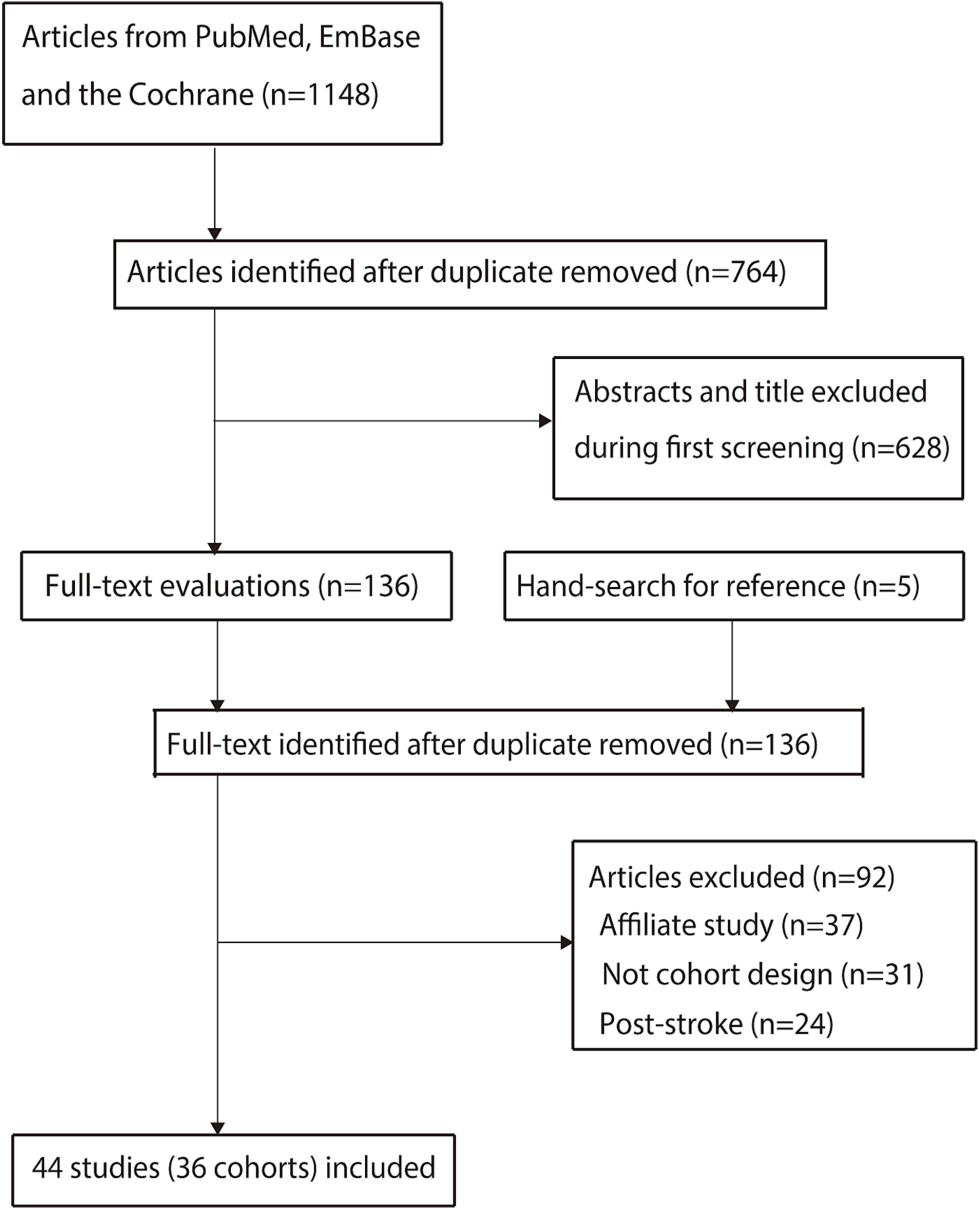
Flowchart of the literature search and study selection.

The study selection process is shown in Figure 1. We initially identified 1,148 articles through the electronic searches, of which 1,012 were excluded after removing duplicates and irrelevant studies. After the full-text review of the remaining 136 articles, 92 articles were excluded, leaving 44 studies. Of these, a total of 36 study cohorts were selected for the final analysis (23–66). Manual searching of the reference lists of these studies did not identify any additional studies meeting the criteria. The general characteristics of the included studies are shown in Table 1, and the outcome ascertainment for each study is summarized in Supplementary material 2.

**Table 1:** The baseline characteristics of the included studies and involved individuals.

Study	Study design	Geographical region	Study period	Sample size	Age (years)	Male (%)	Exposure	Outcomes	Number of stroke	Follow-up (years)	Adjusted factors	NOS
Wassertheil-Smoller 1996 (23)	Prospective	USA	1985–1988	4,367	72.0	47.0	Depression (CES-D)	Stroke	204	4.5	Age, sex, ethnicity, baseline depression, randomization group, years of education,history of stroke, MI, or DM, smoking, baseline ADL, and ADL	7
Simons 1998 (24)	Prospective	Australia	1988–1997	2,805	69.0	44.0	Depression (CES-D)	IS	306	8.2	Lipoprotein, alcohol intake, ECG evidence of left ventricular hypertrophy, and family history of premature CHD	8
Whooley 1998 (25)	Prospective	USA	1986–1990	7,518	71.5	0.0	Depression (GDS)	Stroke mortality	94	7.0	Age, history of MI, stroke, COPD, hypertension, DM, smoking, perceived health, and cognitive function	7
Ostir 2001 (26, 27)	Prospective	USA	1986–1992	2,478/2,812	> 65.0	31.2	Depression (CES-D)	Stroke	340/270	6.0/12.0	Age, income, education, marital status, BMI, smoking, heart attack, DM, and systolic blood pressure	7
Yasuda 2002 (28)	Prospective	Japan	1991–1998	817	72.0	39.0	Depression (30-item GHQ)	Stroke mortality	20	7.5	Age, sex, chronic conditions under treatment, regular PA, and availability of close or casual neighbors	8
Carney 2006 (29)	Retrospective	USA	1996–2001	727,336	37.7	47.5	Schizophrenia (ICD-9)	Stroke	3,201	2.2	Age, sex, residence, and number of non-mental health care visits.	9
Kamphuis 2006 (30)	Prospective	Finland, Italy, and the Netherlands	1984–2000	799	70.0–90.0	100.0	Depression (SDS)	Stroke mortality	66	10.0	Age, country, education, BMI, smoking, alcohol intake, SBP, TC, HDL, and PA	8
Lin 2007 (31–35)	Retrospective	China	1998–2003	18,702	34.0	50.1	Bipolar disorder (ICD-9); Schizophrenia (ICD-9), Depression (ICD-9)	Stroke	315	6.0/5.0	Age, sex, geographic location, hypertension, DM, hyperlipidemia, COPD, renal disease, alcohol dependence, and substance dependence	9
Arbelaez 2007 (36–38)	Prospective	USA	1989–2000	5,525	72.7	41.8	Depression (CES-D)	Stroke, IS	611	11.0	Age, sex, ethnicity, occupation, income, education level, marital status, hypertension, DM, smoking, CHD, TC, HDL, LDL, TG, and BMI	8
Kawamura 2007 (39)	Prospective	Japan	1985–2000	920	74.0	40.1	Depression (SDS)	Stroke mortality	185	15.0	Age	7
Bos 2008 (40)	Prospective	The Netherlands	1997–1999	4,424	72.0	39.8	Depression (CES-D)	Stroke, IS	291	5.6	Age, sex, SBP, DM, cigarette smoking, intima–media thickness, history of MI, history of PTCA or CABG, history of TIA, antithrombotic drug use, antihypertensive drug use, cholesterol-lowering drug use, psycholeptic drug use, and psychoanaleptic drug use	9
Liebetrau 2008 (41)	Retrospective	Sweden	1986–1990	494	85.0	28.9	Depression (DSMMD)	Stroke	56	3.0	Sex	7
Bresee 2010 (42)	Retrospective	Canada	1995–2006	2,310,391	45.3	49.5	Schizophrenia (ICD-9)	Stroke	100,874	12.0	Age, sex, socioeconomic status, and general practitioner visits	8
Peters 2010 (43)	Prospective	UK	1999–2008	2,656	83.4	39.4	Depression (GDS)	Stroke	97	2.1	Age, sex, treatment allocation, country area, educational level, living alone, number of comorbidities, previous cardiovascular disease, previous treatment, and previously diagnosed hypertension	7
Laursen 2011 (44)	Retrospective	Denmark	1995–2006	2,450,812	15.0–52.0	NA	Bipolar disorder (ICD-8)	Stroke	NA	12.0	Sex, age, and calendar time	8
Pan 2011 (45)	Prospective	USA	2000–2006	80,574	66.0	0.0	Depression (MHI-5)	Stroke, IS, HS	1,033	6.0	Age, marital status, parental history of MI, ethnicity, PA level, BMI, alcohol consumption, smoking status, menopausal status, postmenopausal hormone therapy, current aspirin use, current multivitamin use, the Dietary Approaches to Stop Hypertension dietary score, history of hypertension, hypercholesterolemia, DM, cancer, and heart diseases.	9
Majed 2012 (46)	Prospective	France and Northern Ireland	1991–1993	9,601	55.0	100.0	Depression (CES-D)	Stroke	136	10.0	Age, study centers, and socioeconomic factors, including marital status, education level, employment status, PA, smoking status, daily alcohol intake, SBP, use of anti-hypertensive drugs, BMI, TC, HDL, treatment for DM, and use of antidepressant treatment.	8
Westman 2013 (47, 48)	Prospective	Sweden	1987–2006	10,631,208	NA	NA	Bipolar disorder (ICD-8-10), Schizophrenia (ICD-9-10)	Stroke, Stroke mortality	419	20.0	Sex, age, and year of follow-up	8
Köhler 2013 (49)	Prospective	Germany	2003–2004	2,854	> 75.0	34.2	Depression (GDS)	Stroke	209	6.0	Age, sex, marital status, educational level, smoking, hypertension, MI, DM, PAD, TIA, hypercholesterolemia, hyperlipidemia, ApoE status, mobility, IADL impairment, level of alcohol consumption, and MCI status	8
Péquignot 2013 (50)	Prospective	France	1999–2001	7,308	73.0	36.5	Depression (CES-D)	Stroke, Stroke mortality	116	5.3	Age, study center, sex, smoking status, alcohol consumption, hypertension, impaired fasting glycemia or DM, hypercholesterolemia, living alone, education level, and MMSE score	8
Jackson 2013 (51)	Prospective	Australia	1998–2010	10,399	52.5	0.0	Depression (CES-D)	Stroke	177	12.0	Present age, education, homeownership, hypertension, DM, heart disease, hysterectomy/ oophorectomy, smoking, alcohol use, PA, and BMI	8
Gafarov 2013 (52)	Prospective	Russia	1995–2010	870	25.0–64.0	0.0	Depression (MOPSY)	Stroke	35	16.0	Age	7
Everson-Rose 2014 (53)	Prospective	USA	2000–2002	6,749	62.1	47.1	Depression (CES-D)	Stroke	195	8.5	Age, ethnicity, sex, education, study site, SBP, alcohol use, smoking status, moderate and vigorous PA, BMI, height, use of antihypertensives, DM/fasting blood glucose status, HDL, and TG	8
Brunner 2014 (54)	Prospective	UK	1985–2009	31,395	44.4	67.2	Depression (CES-D, 30-item GHQ)	Stroke	168	24.0	Age, sex, and ethnicity	7
Correll 2015 (55)	Retrospective	USA	2006–2010	284,234	44.5	29.4	Schizophrenia (ICD-9)	Stroke	2,027	4.0	Sex, age, psychiatric diagnosis. and medical morbidity count	7
Prieto 2016 (56)	Prospective	USA	1966–2013	668	37.0	48.0	Bipolar disorder (DSMMD)	Stroke	58	18.7	Alcohol use disorder, hypertension, diabetes, and smoking	7
Sun 2016 (57)	Prospective	China	2004–2013	487,377	51.0	40.9	Depression (DSMMD)	Stroke	27,623	7.2	Age, sex, marital status, household income, education, region, smoking status, alcohol consumption, PA, BMI, history of hypertension, and history of DM	9
Gabilondo 2017 (58)	Retrospective	Spain	2007–2010	2,255,406	44.0	49.1	Schizophrenia (ICD-10)	Stroke	55,787	3.0	Age, sex, and deprivation index	8
Zahodne 2017 (59)	Prospective	USA	1994–2003	3,524	74.3	41.8	Depression (CES-D)	Stroke	665	6.4	Age, sex, ethnicity, education, marital status, self-rated health, activities of daily living, PA, cognitive status, BMI, and the Framingham Risk Score	7
Tsai 2017 (60)	Prospective	China	1997–2010	40,645	55.0	31.1	Depression (ICD-9)	Stroke	4,550	8.2	Age, sex, level of urbanization, income, visits seeking medical care, and comorbidity	9
Momen 2020 (61)	Prospective	Denmark	2000–2016	5,940,299	32.1	49.8	Schizophrenia (ICD-8,10)	Stroke	283,217	15.0	Age, sex, calendar time, and previous mental disorders	9
Meza 2020 (62)	Prospective	Mexico	2001–2015	10,720	62.0	45.2	Depression (CES-D)	Stroke	546	11.4	Age, sex, education, marital status, urbanicity, state of residence, income, hypertension, and DM	8
Sico 2021 (63)	Prospective	USA	2003–2014	106,333	48.9	97.0	Depression (ICD-9)	IS	4,355	9.2	Age, sex, ethnicity, SBP, DBP, LDL, HDL, TG, statin use, DM, BMI, smoking status, eGFR, hemoglobin, hepatitis C infection, atrial fibrillation, viral load, CD4 count, antiretroviral therapy, alcohol use, cocaine use, and antidepressant medication use	9
Cui 2021 (64)	Prospective	China	2013–2018	8,491	59.4	44.8	Depression (CES-D)	Stroke	545	5.0	Age, sex, education, marital status, place of residence, smoking status, drinking frequency, BMI, hypertension, DM, and heart disease	8
Ford 2021 (65)	Prospective	USA	2003–2007	24,045	64.5	45.0	Depression (CES-D)	Stroke	1,262	9.2	Age, ethnicity, sex, hypertension, DM, smoking, atrial fibrillation, heart disease, left ventricular hypertrophy, education, income, and social network	8
Shen 2022 (66)	Prospective	USA	2005–2018	34,079	20.0–85.0	49.4	Depression (PHQ-9)	Stroke	1,149	13.0	Age, sex, ethnicity, marital status, family poverty income ratio, education, smoking status, and BMI	7

*BMI, body mass index; CABG, coronary artery bypass graft; CES-D, Center for epidemiological studies-depression; CHD, coronary heart disease; COPD, chronic obstructive pulmonary disease; DM, diabetes mellitus; DSMMD, diagnostic and statistical manual of mental disorders; GDS, Geriatric Depression Scale; HDL, high-density lipoprotein; HS, hemorrhagic stroke; IADL, instrumental activities of daily living; IS, ischemic stroke; LDL, low-density lipoprotein; MCI, mild cognitive impairment; MHI-5, 5-item Mental Health Index; MI, myocardial infraction; MMSE, Mini–Mental State Examination; PA, physical activity; PAD, peripheral artery disease; PHQ-9, nine-item Patient Health Questionnaire; PTCA, percutaneous transluminal angioplasty; SBP, systolic blood pressure; SDS, self-rating Depression Scale; TC, total cholesterol; TG, triglycerides; TIA, transient ischemic attack.

### Study characteristics

Of the 36 included cohorts, 29 were prospective, while the remaining seven were retrospective. These studies involved a total of 25,519,635 individuals, and the sample size ranged from 494 to 10,631,208. Moreover, the follow-up duration ranged from 2.1 to 24.0 years. A total of 29 cohorts were performed in Western countries, while the remaining seven studies were conducted in Eastern countries. The quality of the studies was assessed using the NOS scoring system, and all included studies were of high quality. Among them, eight studies scored 9 stars, 16 studies scored 8 stars, and the remaining 12 studies scored 7 stars.

### Depression

**Figure 2:**
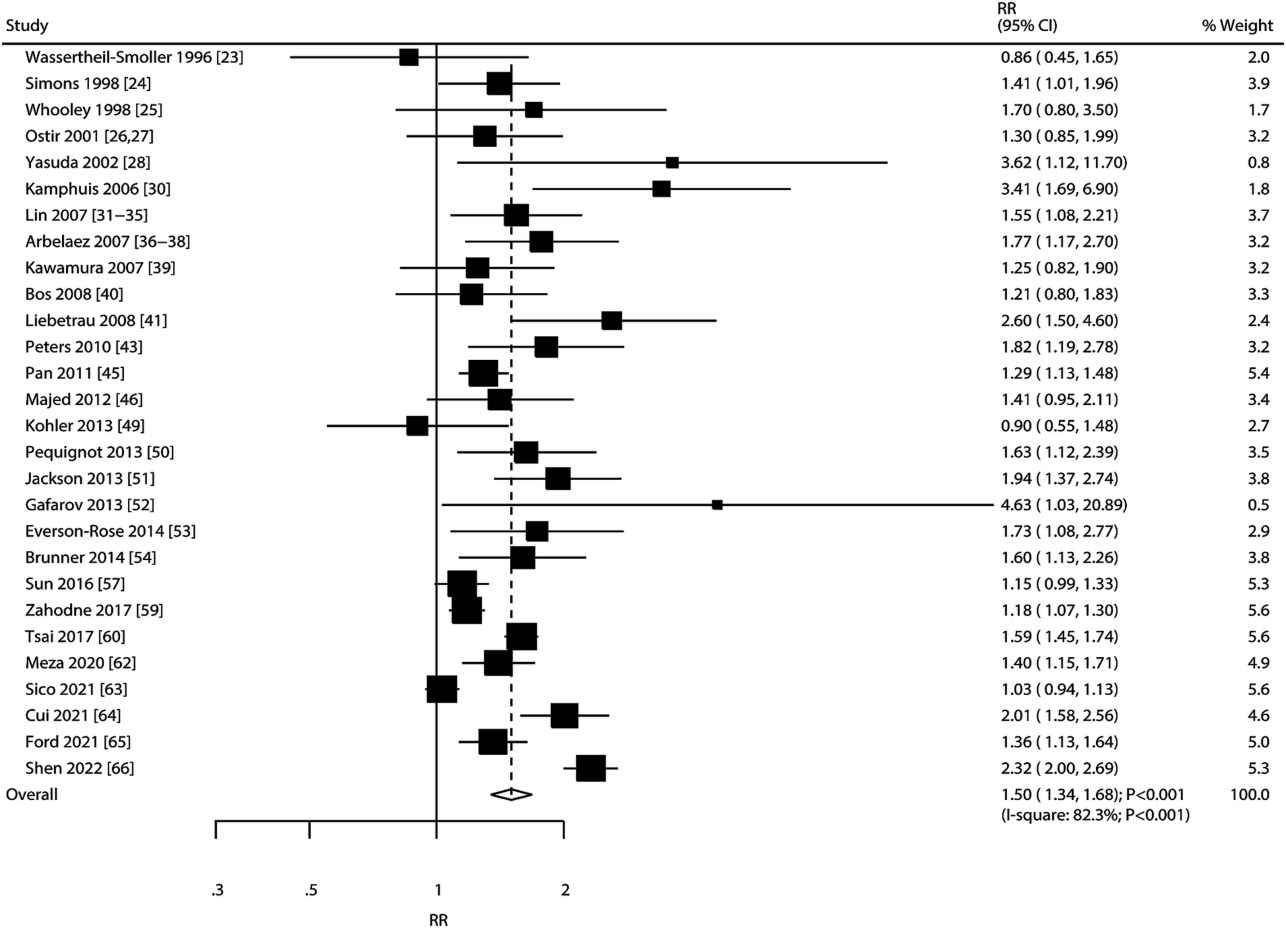
Association between depression and the risk of stroke.

A total of 28 cohorts reported an association between depression and the risk of stroke. The summary RR indicated that depression was associated with an elevated risk of stroke (RR: 1.50; 95% CI: 1.34–1.68; *p* < 0.001), and significant heterogeneity was observed across the included studies (*I^2^* = 82.3%; *p* < 0.001) (Figure 2). The sensitivity analysis indicated that the pooled conclusion for the relationship between depression and stroke risk remained stable after sequentially removing each study (Supplementary material 3 [Figure S1]). The subgroup analyses found that depression was associated with an elevated risk of stroke in most subsets, while no significant association was observed between depression and hemorrhagic stroke (Table 2). Furthermore, we found that the association between depression and stroke was significantly weaker in the subgroup with fully adjusted results compared to the subgroup with generally adjusted results (RR ratio: 0.77; 95% CI: 0.61–0.98; *p* = 0.034). There was no significant publication bias for the association between depression and the risk of stroke (*p*-value for the Egger’s test: 0.074; *p*-value for the Begg’s: 0.221; Supplementary material 4 [Figure S1]).

**Table 2:** Subgroup analyses of the association between the psychiatric disorders and stroke risk.

Psychiatric disorders	Factors	Subgroups	RR and 95% CI	*p*-value	*I^2^* (%)	Q statistic	Ratio of the RR between the subgroups
Depression	Study design	Prospective	1.48 (1.32–1.66)	< 0.001	83.0	< 0.001	0.77 (0.46–1.30); *p* = 0.331
		Retrospective	1.91 (1.16–3.16)	0.011	57.4	0.126	
	Geographical region	Eastern	1.56 (1.26–1.92)	< 0.001	75.7	< 0.001	1.05 (0.82–1.34); *p* = 0.719
		Western	1.49 (1.30–1.70)	< 0.001	83.2	< 0.001	
	Mean age (years)	≥ 70.0	1.48 (1.23–1.79)	< 0.001	59.8	0.003	0.97 (0.77–1.24); *p* = 0.827
		< 70.0	1.52 (1.31–1.76)	< 0.001	88.3	< 0.001	
	Sex	Male	1.88 (1.21–2.94)	0.005	79.2	< 0.001	1.17 (0.70–1.95); *p* = 0.554
		Female	1.61 (1.25–2.09)	< 0.001	80.1	< 0.001	
	Stroke type	Ischemic	1.15 (1.03–1.30)	0.018	42.4	0.122	0.95 (0.73–1.24); *p* = 0.707
		Hemorrhagic	1.21 (0.95–1.53)	0.126	0.0	0.634	
	Outcome definition	Incidence	1.47 (1.31–1.66)	< 0.001	84.0	< 0.001	0.72 (0.46–1.13); *p* = 0.151
		Mortality	2.05 (1.32–3.17)	0.001	49.4	0.095	
	Follow-up (years)	≥ 10.0	1.72 (1.41–2.10)	< 0.001	69.3	0.001	1.24 (0.98–1.56); *p* = 0.071
		< 10.0	1.39 (1.24–1.57)	< 0.001	79.2	< 0.001	
	Adjusted level	Fully	1.39 (1.25–1.55)	< 0.001	70.9	< 0.001	0.77 (0.61–0.98); *p* = 0.034
		General	1.80 (1.45–2.22)	< 0.001	74.5	< 0.001	
Schizophrenia	Study design	Prospective	1.83 (1.08–3.11)	0.025	99.6	< 0.001	1.08 (0.57–2.02); *p* = 0.819
		Retrospective	1.70 (1.21–2.41)	0.003	93.5	< 0.001	
	Geographical region	Eastern	2.02 (1.57–2.60)	< 0.001	-	-	1.19 (0.82–1.72); *p* = 0.360
		Western	1.70 (1.30–2.23)	< 0.001	99.0	< 0.001	
	Mean age (years)	≥ 70.0	-	-	-	-	-
		< 70.0	1.64 (1.26–2.13)	< 0.001	98.5	< 0.001	
	Sex	Male	2.11 (1.38–3.22)	0.001	83.3	0.014	0.91 (0.59–1.39); *p* = 0.652
		Female	2.33 (2.15–2.52)	< 0.001	0.0	0.338	
	Outcome definition	Incidence	1.64 (1.26–2.13)	< 0.001	98.5	< 0.001	0.68 (0.52–0.90); *p* = 0.006
		Mortality	2.40 (2.25–2.55)	< 0.001	-	-	
	Follow-up (years)	≥ 10.0	1.94 (1.35–2.77)	< 0.001	99.6	< 0.001	1.24 (0.74–2.06); *p* = 0.419
		< 10.0	1.57 (1.09–2.27)	0.015	82.6	0.001	
	Adjusted level	Fully	1.58 (1.22–2.05)	< 0.001	74.9	0.019	0.84 (0.60–1.20); *p* = 0.342
		General	1.87 (1.48–2.35)	< 0.001	95.8	< 0.001	
Bipolar disorder	Study design	Prospective	1.38 (1.26–1.52)	< 0.001	0.0	0.578	0.68 (0.55–0.84); *p* < 0.001
		Retrospective	2.02 (1.67–2.44)	< 0.001	0.0	0.927	
	Geographical region	Eastern	2.05 (1.43–2.93)	< 0.001	-	-	1.32 (0.83–2.11); *p* = 0.240
		Western	1.55 (1.15–2.09)	0.004	78.8	0.009	
	Mean age (years)	≥ 70.0	-	-	-	-	-
		< 70.0	1.86 (1.45–2.39)	< 0.001	35.5	0.212	
	Sex	Male	1.32 (1.14–1.53)	< 0.001	-	-	0.92 (0.76–1.12); *p* = 0.411
		Female	1.43 (1.27–1.62)	< 0.001	-	-	
	Outcome definition	Incidence	1.65 (1.27–2.14)	< 0.001	76.3	0.005	0.96 (0.61–1.50); *p* = 0.855
		Mortality	1.72 (1.20–2.48)	0.003	74.6	0.047	
	Follow-up (years)	≥ 10.0	1.55 (1.15–2.09)	0.004	78.8	0.009	0.76 (0.47–1.21); *p* = 0.240
		< 10.0	2.05 (1.43–2.93)	< 0.001	-	-	
	Adjusted level	Fully	2.05 (1.43–2.93)	< 0.001	-	-	1.32 (0.83–2.11); *p* = 0.240
		General	1.55 (1.15–2.09)	0.004	78.8	0.009	

### Schizophrenia

**Figure 3:**
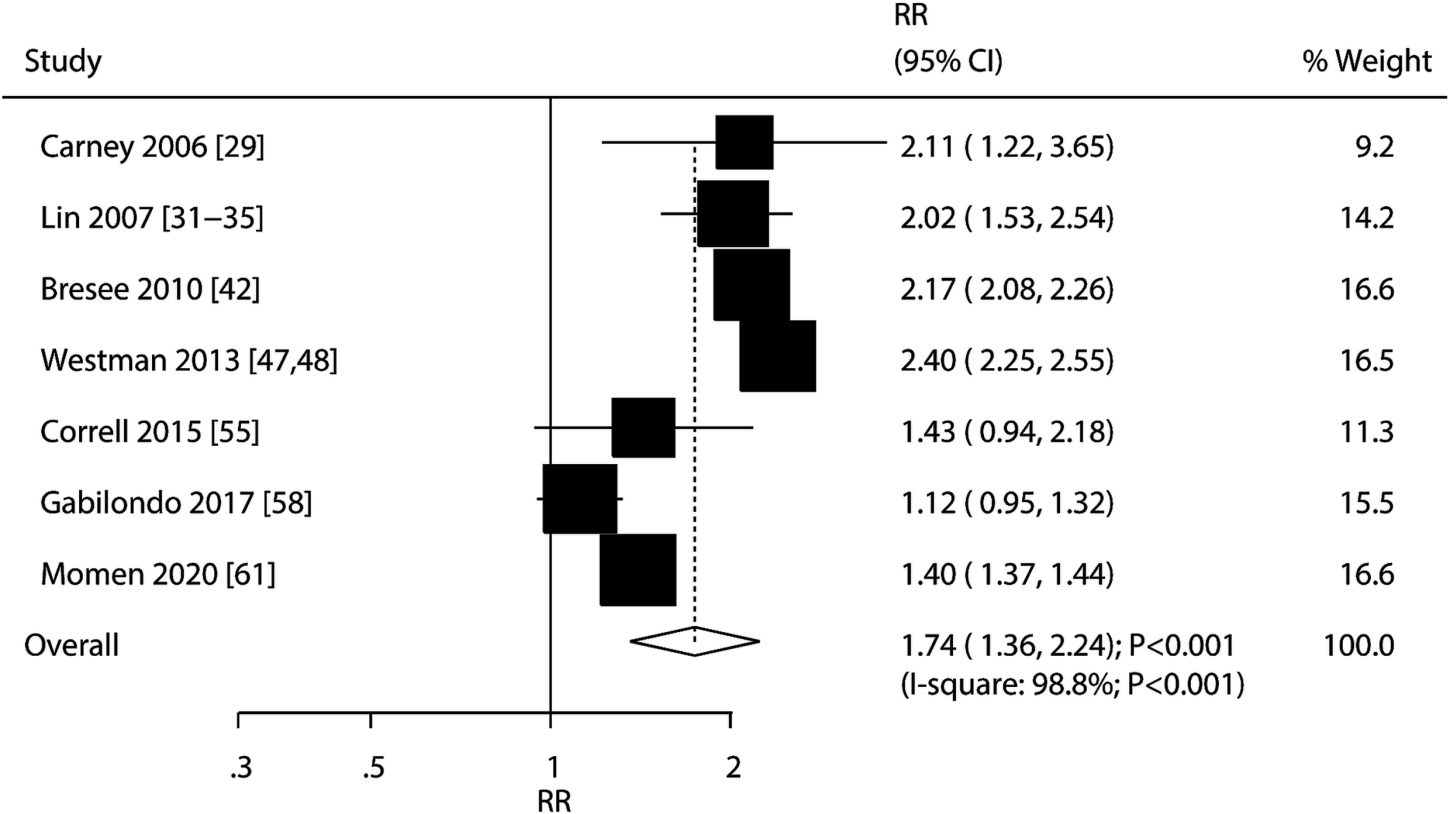
Association between schizophrenia and the risk of stroke.

A total of seven cohorts reported an association between schizophrenia and the risk of stroke. We found that schizophrenia was associated with an elevated risk of stroke (RR: 1.74; 95% CI: 1.36–2.24; *p* < 0.001), and significant heterogeneity was observed among the included studies (*I^2^* = 98.8%; *p* < 0.001) (Figure 3). The sensitivity analysis indicated that the pooled conclusion was robust and not altered by the removal of any particular study (Supplementary material 3 [Figure S2]). The results of the subgroup analyses were consistent with those of the overall analysis, and the relationship between schizophrenia and stroke risk remained significant (Table 2). Moreover, the strength of the association between schizophrenia and the risk of stroke incidence was weaker than that of the association between schizophrenia and the risk of stroke mortality (RR ratio: 0.68; 95% CI: 0.52–0.90; *p* = 0.006). No significant publication bias was observed for the association between schizophrenia and the risk of stroke (*p*-value for the Egger’s: 0.584; *p*-value for the Begg’s test: 1.000; Supplementary material 4 [Figure S2]).

### Bipolar disorder

**Figure 4:**
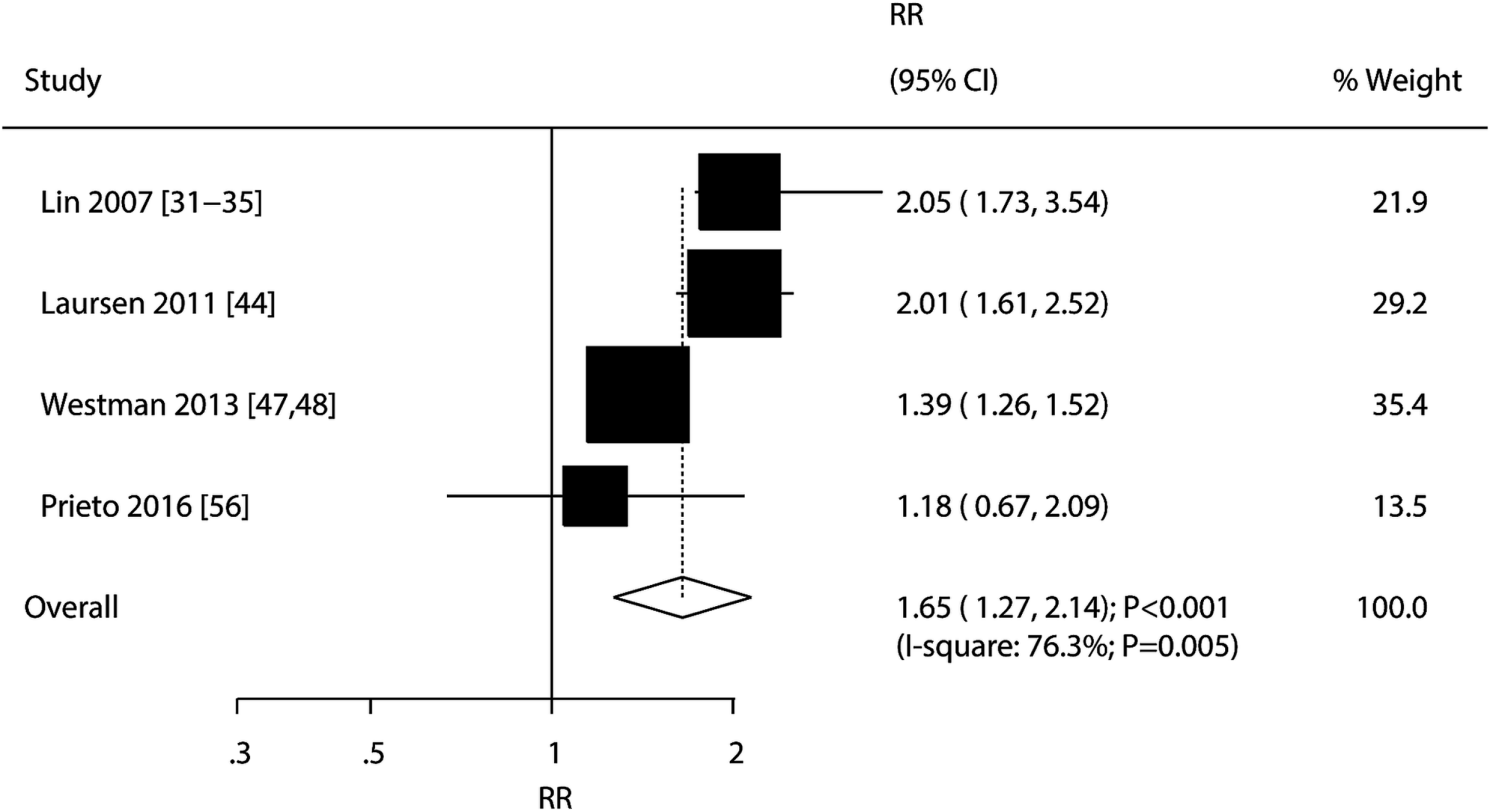
Association between bipolar disorder and the risk of stroke.

A total of four cohorts reported an association between bipolar disorder and the risk of stroke. The summary result indicated that bipolar disorder was associated with an elevated risk of stroke (RR: 1.65; 95% CI: 1.27–2.14; *p* < 0.001), and significant heterogeneity was detected across the included studies (*I^2^* = 76.3%%; *p* = 0.005) (Figure 4). The pooled conclusion remained stable after sequentially removing each study (Supplementary material 3 [Figure S3]). The subgroup analyses found that bipolar disorder was associated with an elevated risk of stroke in all subsets, and the strength of the association between bipolar disorder and the risk of stroke in the prospective cohort studies was weaker than that in the retrospective cohort studies (RR ratio: 0.68; 95% CI: 0.55–0.84; *p* < 0.001; Table 2). There was no significant publication bias for the association between bipolar disorder and the risk of stroke (*p*-value for the Egger’s test: 0.473; *p*-value for the Begg’s test: 1.000; Supplementary material 4 [Figure S3]).

## Discussion

In this meta-analysis involving 25,519,635 participants across 36 study cohorts, we found a significant increase in the risk of stroke associated with depression, schizophrenia, and bipolar disorder. Furthermore, this association persisted in almost all subgroups and remained statistically significant across different study designs and participant characteristics. In addition, the exploratory analysis results indicated that the association between depression and stroke differed when stratified by the adjusted level, and there were significant differences in the association between schizophrenia and the incidence and mortality of stroke. Finally, the association between bipolar disorder and the risk of stroke differed based on the study design.

Several systematic reviews and meta-analyses have already addressed the association between psychiatric disorders and the risk of stroke (67–69). Cai et al. conducted a meta-analysis of 17 cohort studies and found a positive association between depression and the risk of stroke. However, the exploratory analysis was not comprehensive, and the comparison results between the subgroups were not fully elaborated (67). Chu et al. identified 20 studies and found that schizophrenia was significantly associated with an increased risk of stroke morbidity and mortality (68). However, the majority of the studies included were not cohort studies, so they could not confirm a causal relationship between schizophrenia and the risk of stroke. Yuan et al. performed a meta-analysis of seven studies (six cohort studies and one case–control study) and found that bipolar disorder was associated with an elevated risk of both stroke morbidity and mortality (69). The analysis of this study was not based on cohort studies, and multiple studies reported on the same cohort, so the pooled conclusions might have been overestimated and could not prove a causal relationship between bipolar disorder and the risk of stroke. Therefore, the current study was performed to comprehensively illustrate the association between psychiatric disorders and the risk of stroke.

While our study provides valuable insights into the relationship between psychiatric disorders and stroke, several potential confounding factors could pose a threat to the causality of our findings. It is important to consider the following factors when interpreting the results: (1) older age and certain sex differences can influence both the presence of psychiatric disorders and the risk of stroke; (2) socioeconomic status, including income, education, and occupation, can confound the observed associations; (3) smoking and alcohol consumption are known risk factors for stroke and can also be associated with psychiatric disorders; (4) pre-existing medical conditions such as hypertension, diabetes, and cardiovascular disease can affect both the risk of psychiatric disorders and the risk of stroke; and (5) the use of certain medications, particularly those used to manage psychiatric disorders, can also confound the observed relationships.

The study found that the risk of stroke was significantly elevated in patients with depression, and this combined result was highly stable and consistent with those of previous meta-analyses (67). Several reasons could explain the significant association between depression and the risk of stroke: (1) patients with depression may experience physiological changes, such as increased sympathetic nervous system activity, elevated heart rate, and high blood pressure due to long-term exposure to stress, which could potentially increase the risk of stroke (70); (2) patients with depression may have increased platelet activity, leading to a higher risk of thrombosis and, consequently, an increased likelihood of stroke occurrence (71); (3) depression is associated with chronic inflammation and immune system dysfunction, and these inflammatory factors may impair vascular endothelial function, promote the development of atherosclerosis, and increase the risk of stroke (72, 73); (4) patients with depression are more likely to engage in unhealthy lifestyle choices, such as poor dietary habits, lack of exercise, smoking, and excessive drinking, which can increase the risk of stroke (74); and (5) the effects of antidepressants on metabolic parameters, including weight gain, hypertension, and hyperlipidemia, can promote the development of atherosclerosis and other cardiovascular diseases through various mechanisms, thereby increasing the risk of stroke. Specifically, weight gain can lead to obesity, which, in turn, triggers insulin resistance and inflammatory responses; hypertension damages the vascular endothelium, promoting arterial wall hardening and plaque formation; and hyperlipidemia increases cholesterol and triglyceride levels in the blood, accelerating the progression of atherosclerosis. These factors collectively contribute to an increased risk of stroke (75, 76). Furthermore, we found that the association between depression and the risk of stroke differed when stratified by the adjusted level. This could be due to the influence of the unadjusted confounders, which might have led to inaccuracies in the conclusions. Finally, the choice of confounding factors may also affect the detection of potential effect modification in study results. If a confounding factor itself interacts with exposure and outcome variables, adjusting for it may mask the presence of such interaction.

We found that schizophrenia was associated with an elevated risk of stroke. The main reasons for this result include the following: (1) Patients with schizophrenia often receive antipsychotic medications, which may increase the risk of cardiovascular diseases, including stroke. Some antipsychotic drugs, particularly second-generation or atypical antipsychotics, are known to have significant metabolic side effects that can contribute to these risks. Specifically, these medications can lead to several risk factors for cardiovascular diseases, including weight gain, hypertension, hyperlipidemia, insulin resistance and diabetes, and inflammatory markers (77). (2) Patients with schizophrenia often face issues such as sleep problems, poor dietary habits, lack of exercise, and other unhealthy lifestyle factors, which can also increase the risk of stroke (78). (3) Schizophrenia may be related to the patient’s physiological and psychological state, which may lead to increased inflammatory responses and abnormal stress responses, thereby increasing the risk of stroke (79). Furthermore, the relationship between schizophrenia and stroke risk was found to differ according to the outcome definition, which could be explained by the fact that the stroke mortality rate is significantly lower than the overall stroke incidence, which could have led to poor stability of the results.

The summary results indicated that bipolar disorder was associated with an elevated risk of stroke. The specific mechanisms that may contribute to the increased risk of stroke in patients with bipolar disorder include the following: (1) Vascular dysfunction: Patients with bipolar disorder may have vascular abnormalities, such as endothelial dysfunction and a tendency for thrombus formation, which can increase the risk of stroke (80). (2) Inflammatory response: Previous studies suggest that patients with bipolar disorder often exhibit chronic low-grade inflammation, and the release of inflammatory factors may promote atherosclerosis development, thereby increasing the risk of stroke (81). (3) Autonomic nervous system dysregulation: Patients with bipolar disorder may experience dysregulation of the autonomic nervous system, leading to abnormalities in blood pressure, heart rate, and other physiological parameters, which can elevate the risk of stroke (82). (4) Treatment-related adverse events: The medications used to treat bipolar disorder can have side effects that affect various physiological parameters, including blood coagulation, blood pressure, lipid levels, and other factors, thereby increasing the risk of stroke (83).

There are several shortcomings of this study that warrant acknowledgment: (1) Given its reliance on both prospective and retrospective cohort studies, our findings might have been susceptible to uncontrolled recall biases and confounding influences. (2) Variations in the psychiatric disorder assessment criteria across different studies could have potentially skewed the evaluation of disease severity. (3) Although our analysis excluded other common psychiatric disorders, such as anxiety, the role of anxiety in stroke risk remains an important area for future research. (4) Inconsistencies in the covariates adjusted for in the reported effect sizes across the studies undermined the precision of establishing a connection between psychiatric disorders and stroke risk. (5) Not all studies provided detailed data on medication use, which prevented us from fully controlling for medication use when synthesizing the results. (6) The inherent constraints of a meta-analysis based solely on published literature, such as the exclusion of unpublished data and reliance on pooled data, hinder more nuanced and detailed analyses.

## Conclusion

This study found an association between psychiatric disorders—depression, schizophrenia, and bipolar disorder—and an increased risk of stroke. Individuals with these disorders should adopt enhanced stroke prevention strategies to improve patient outcomes.

## Data Availability

The original contributions presented in the study are included in the article/Supplementary material, further inquiries can be directed to the corresponding author.
