# Supplementary material for: Association between psychiatric disorders and the risk of stroke: a meta-analysis of cohort studies
Source: Front Neurol. 2024 Dec 18;15:1444862. doi: 10.3389/fneur.2024.1444862 (PMC11688293; doi:10.3389/fneur.2024.1444862)
Supplement: Supplementary file 1 [file Table_1.DOCX]

Table S1. The outcome ascertainment of included studies

| Study | Outcome ascertainment |
| --- | --- |
| Wassertheil-Smoller 1996 [23] | Nonfatal stroke was defined as the rapid onset of new neurologic deficit attributed to obstruction or rupture in the arterial system and persisting for at least 24 hours unless death supervened. Fatal stroke was established from death certificates or autopsy reports and included preterminal hospitalization data. |
| Simons 1998 [24] | Stroke outcomes were ascertained exclusively by review of hospital and death records (the latter used only in case of death outside hospital) |
| Whooley 1998 [25] | Cerebrovascular disease (ICD-9 codes 430-438) |
| Ostir 2001 [26,27] | Cerebrovascular disease (ICD-9 codes 430-438) |
| Yasuda 2002 [28] | Cerebrovascular disease (ICD-9 codes 430-438) |
| Carney 2006 [29] | Inpatient and outpatient claims data |
| Kamphuis 2006 [30] | Cerebrovascular disease (ICD-9 codes 430-438) |
| Lin 2007 [31-35] | Cerebrovascular disease (ICD-9 codes 430-438) |
| Arbelaez 2007 [36-38] | Cerebrovascular disease (ICD-9 codes 430-438) |
| Kawamura 2007 [39] | Cerebrovascular disease (ICD-9 codes 430-438) |
| Bos 2008 [40] | Cerebrovascular disease (ICD-10 codes 161, 163, 164) |
| Liebetrau 2008 [41] | Cerebrovascular disease (ICD-9 codes 430-438) |
| Bresee 2010 [42] | Cerebrovascular disease (ICD-9 codes 430-438; ICD-10 codes 160, 169, G45) |
| Peters 2010 [43] | The trial investigators and validated by an independent endpoint committee blinded to trial treatment |
| Laursen 2011 [44] | Cerebrovascular disease (ICD-10 codes 161, 163, 164) |
| Pan 2011 [45] | National Survey of Stroke criteria |
| Majed 2012 [46] | World Health Organization MONICA criteria, as a new focal or global neurological deficit with a rapid onset and of vascular origin, persisting for more than 24 hours |
| Westman 2013 [47,48] | Cerebrovascular disease (ICD-9 codes 430-438; ICD-10 codes 160-169) |
| Köhler 2013 [49] | General practitioner filled out a questionnaire asking for the presence |
| Péquignot 2013 [50] | World Health Organization, as a new focal neurological deficit of sudden or rapid onset, of presumed vascular origin that lasted 24 h or more, or that lead to death |
| Jackson 2013 [51] | Cerebrovascular disease (ICD-10 codes I60–I60.9, I61.0–I61.9, I63.0–I63.9, and I64) |
| Gafarov 2013 [52] | World Health Organization MONICA criteria |
| Everson-Rose 2014 [53] | Stroke was defined as rapid onset of documented focal neurological deficits lasting 24 hours or until death and, if  <24 hours, with imaging evidence (typically computed tomography or MRI) of a clinically relevant lesion |
| Brunner 2014 [54] | Cerebrovascular disease (ICD-9 codes 430-438; ICD-10 codes 160-169) |
| Correll 2015 [55] | Cerebrovascular disease (ICD-9 codes 434, 438) |
| Prieto 2016 [56] | Cerebrovascular disease (ICD-9 codes 430-438) and imaging stroke criteria |
| Sun 2016 [57] | Cerebrovascular disease (ICD-10 codes 160, 161, 163, 164) |
| Gabilondo 2017 [58] | Cerebrovascular disease (ICD-10 codes 160, 161, 163, 164) |
| Zahodne 2017 [59] | Cerebrovascular disease (ICD-9 codes 430-438) |
| Tsai 2017 [60] | Cerebrovascular disease (ICD-9 codes 430-438) |
| Momen 2020 [61] | Cerebrovascular disease (ICD-10 codes 160, 161, 163, 164) |
| Meza 2020 [62] | Incident doctor-diagnosed strokes were self-reported at each follow-up interview |
| Sico 2021 [63] | Cerebrovascular disease (ICD-9 codes 430-438) |
| Cui 2021 [64] | Self- or proxy-reported doctor diagnosis |
| Ford 2021 [65] | World Health Organization-defined stroke or clinical stroke meeting the American Heart Association criteria |
| Shen 2022 [66] | Participants who answered “yes” to the question “has a doctor or other health professional ever told you that you had CHD/stroke?” on the medical conditions section of the household questionnaire through home interview were considered to have CHD/stroke |
