# Supplementary material for: Association between psychiatric disorders and the risk of stroke: a meta-analysis of cohort studies
Source: Front Neurol. 2024 Dec 18;15:1444862. doi: 10.3389/fneur.2024.1444862 (PMC11688293; doi:10.3389/fneur.2024.1444862)
Supplement: Supplementary file 2 [file Data_Sheet_1.DOCX]

**Search strategy in PubMed:**

("bipolar disorder"[MeSH Terms] OR "bipolar disorder"[All Fields]) OR ("schizophrenia"[MeSH Terms] OR "schizophrenia"[All Fields]) OR ("depression"[MeSH Terms] OR "depression"[All Fields]) AND ("stroke"[MeSH Terms] OR "stroke"[All Fields] OR "cerebrovascular accident"[MeSH Terms] OR "cerebrovascular accident"[All Fields]) AND ("risk"[MeSH Terms] OR "risk"[All Fields] OR "risk factors"[MeSH Terms] OR "risk factors"[All Fields])

**Search strategy in EmBase:**

('bipolar disorder'/exp OR 'bipolar disorder') OR ('schizophrenia'/exp OR 'schizophrenia') OR ('depression'/exp OR 'depression') AND ('stroke'/exp OR 'stroke' OR 'cerebrovascular accident'/exp OR 'cerebrovascular accident') AND ('risk factor'/exp OR 'risk factor' OR 'risk'/exp OR 'risk')

**Search strategy in Cochrane library:**

(bipolar disorder OR schizophrenia OR depression) AND (stroke risk OR "risk of stroke")
