# Supplementary material for: Association between psychiatric disorders and the risk of stroke: a meta-analysis of cohort studies
Source: Front Neurol. 2024 Dec 18;15:1444862. doi: 10.3389/fneur.2024.1444862 (PMC11688293; doi:10.3389/fneur.2024.1444862)
Supplement: Supplementary file 3 [file Data_Sheet_2.DOCX]

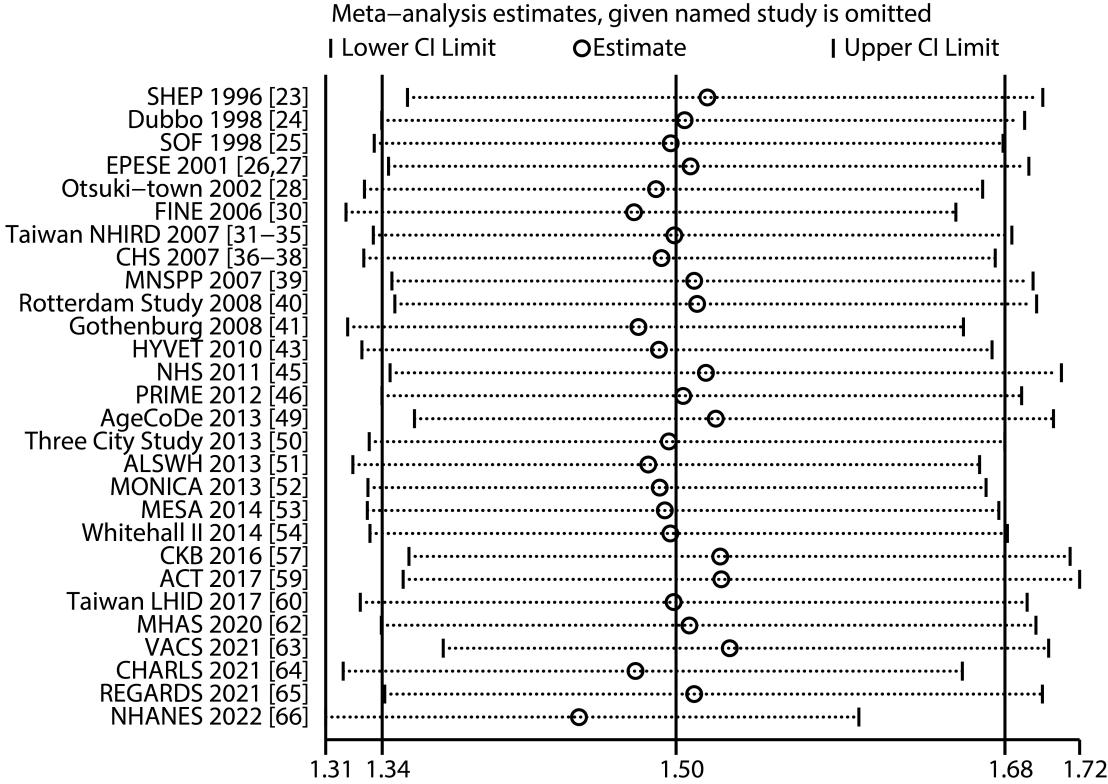


Figure S1. Sensitivity analysis for the association of depression with the risk of stroke


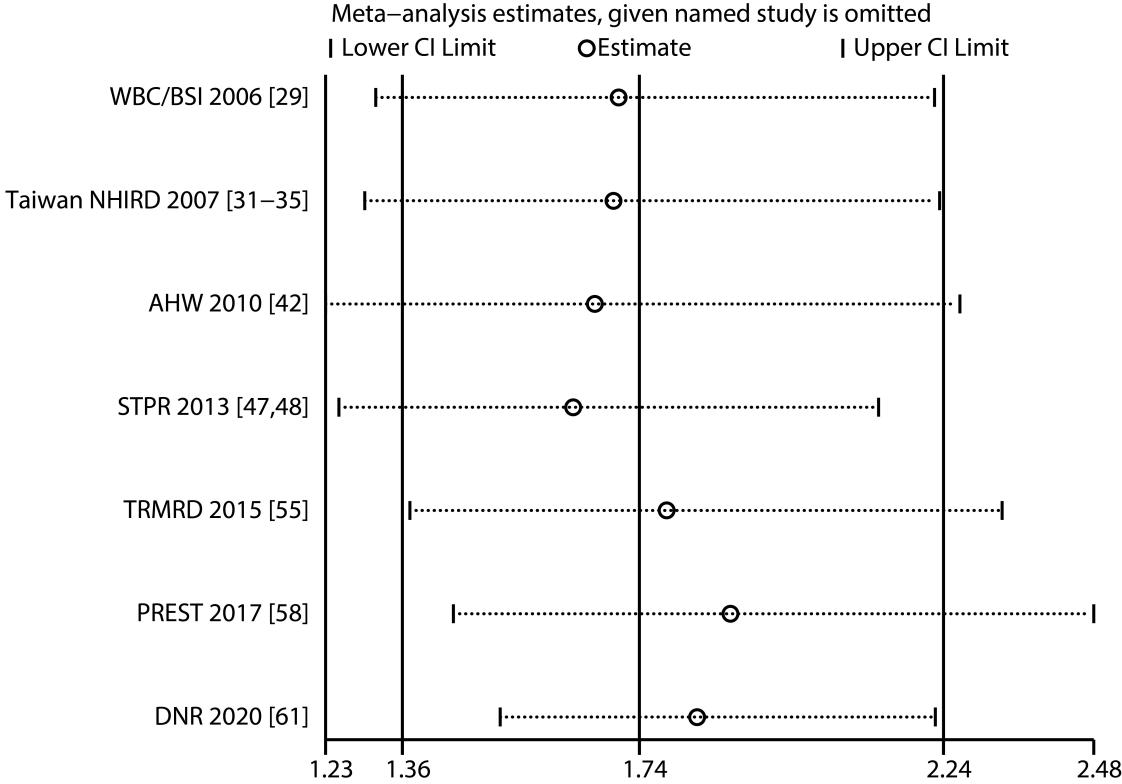


Figure S2. Sensitivity analysis for the association of schizophrenia with the risk of stroke


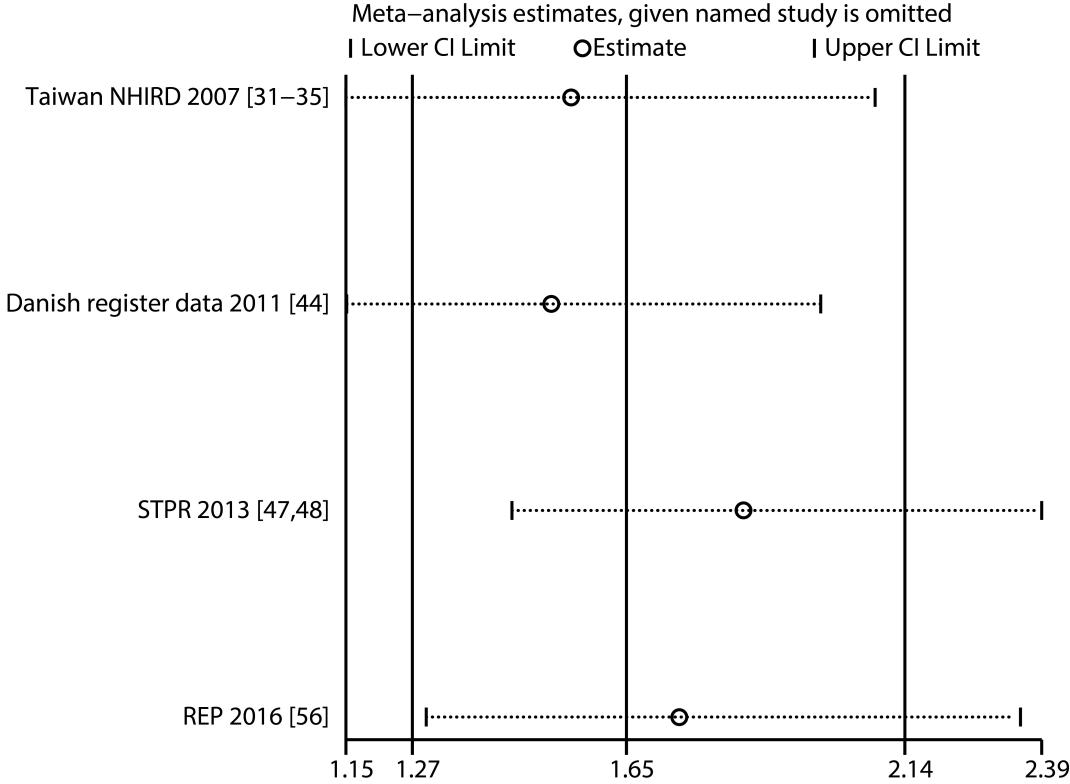


Figure S3. Sensitivity analysis for the association of bipolar disorder with the risk of stroke
